# P-1829. Genomic Characterization of Clinically Significant Isolates and Its Effects on Clinical Outcomes, 2022 to 2024

**DOI:** 10.1093/ofid/ofae631.1992

**Published:** 2025-01-29

**Authors:** Deisy Contreras, Jonathan Grein, Margie A Morgan

**Affiliations:** Cedars-Sinai Medical Center, Los Angeles, California; Cedars-Sinai Medical Center, Los Angeles, California; Cedars-Sinai Medical Center, Los Angeles, California

## Abstract

**Background:**

Antimicrobial resistance has evolved into a complex global issue involving many clinically relevant organisms. Among these pathogens, carbapenem-resistant organisms (CROs) are of particular concern due to their limited treatment options and high mortality rates. In collaboration with Solu, whole-genome sequencing (WGS) was performed on clinical isolates recovered from our patient population to gain a better insight into resistance mechanisms present and their direct effect on clinical outcomes.

**Methods:**

For genomic characterization (WGS), a total of 109 carbapenem resistant isolates across three clinically relevant bacterial groups (*Enterobacterales, Pseudomonas aeruginosa and Acinetobacter baumannii complex)* were sequenced. Solu utilized a hybrid assembly approach utilizing both long reads generated with Oxford Nanopore Technologies (ONT) complemented by Illumina short reads for the reconstruction of bacterial genomes.

**Results:**

Multilocus sequence typing (MLST) revealed genetic diversity among all three groups of bacterial isolates. Among members of the family *Enterobacterales,* there were eight distinct sequence types: ST-131, ST-1431, ST-167, ST-2155, ST-361, ST-410, ST-685 and ST-405. *A. baumannii complex* strains mostly comprised of ST-2 and ST-1022. The presence of numerous sequence types was observed with the *P. aeruginosa* isolates, highlighting the biocomplexity within our patient population. Furthermore, genomic analysis of these isolates disclosed a broad range of antimicrobial resistance genes that span across several classes of antibiotics. Isolates across all genera harbored plasmid-mediated carbapenemase genes *bla-NDM-5, bla-NDM1, bla-OXA-48* and *bla-OXA23.* Interestingly, cefiderocol resistance was primarily associated with the *Escherichia coli* ST-405 strain containing AMR gene *bla-NDM5.* Patients associated with a carbapenemase-producing strain were observed to have higher mortality rates (12.8%) and longer length of hospital stay (25.6%).

**Conclusion:**

By having a comprehensive insight into the genomic composition of isolates that make up our patient population, we can provide a more targeted medical treatment approach and investigate and better prevent transmission events.

**Disclosures:**

**All Authors**: No reported disclosures

